# Typical Electron-Withdrawing Groups Are *ortho*, *meta*-Directors Rather than *meta*-Directors in Electrophilic Aromatic Substitution

**DOI:** 10.1021/acs.joc.5c00426

**Published:** 2025-04-22

**Authors:** Paul R. Rablen

**Affiliations:** Department of Chemistry and Biochemistry, Swarthmore College, 500 College Ave., Swarthmore, Pennsylvania 19081, United States

## Abstract

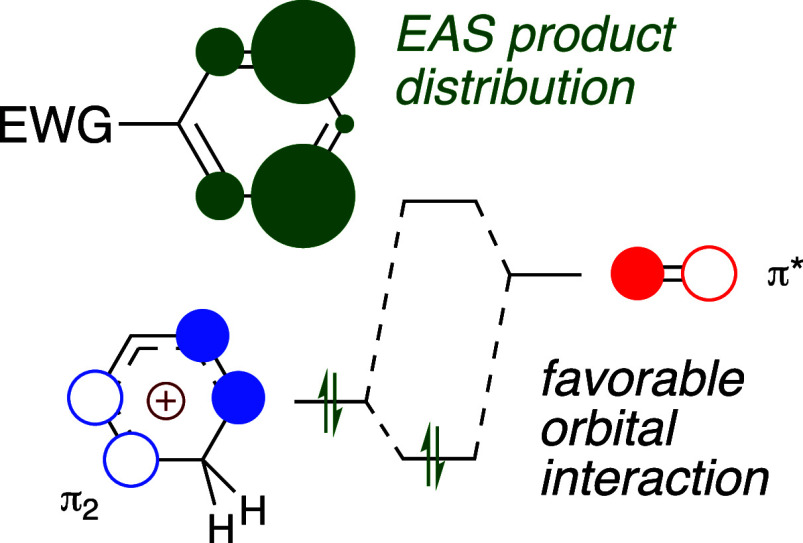

Electron-withdrawing groups are traditionally considered *meta*-directing in aromatic substitution reactions. However, when the pre-existing substituent is a π-acceptor, both experiment and calculation indicate that substantial amounts of *ortho* as well as *meta* substitution occur, with very little *para* reactivity. A simple perturbative MO argument rationalizes this finding. It is therefore suggested that these substituents are best understood as *ortho*, *meta*-directors, with a preference for *meta*, just as electron-donating groups are considered *ortho*, *para*-directors, with a preference for *para*.

The electrophilic aromatic substitution (EAS) reaction has been studied extensively for many decades, and it is far beyond the scope of this Note to provide even a cursory review. The mechanism typically proceeds through a cyclohexadienyl cation intermediate in which the electrophile has added to the aromatic ring, or at least a transition structure that resembles such an intermediate, although many exceptions certainly exist. This intermediate, often referred to as a σ-complex or, in the older literature, as a Wheland intermediate, plays a central role in explaining the behavior of the reaction, particularly with regard to how a pre-existing substituent influences reactivity and product regiochemistry.^1−3^

Pre-existing substituents on a benzene ring are traditionally divided into three categories depending on how they affect subsequent EAS reactions. Most electron-donating groups (EDGs) are categorized as *ortho*, *para*-directing activators (OPAs), since they accelerate the reaction and yield *ortho*- and *para*-substituted products in strong preference to *meta*-substituted products. This category includes alkyl groups as well as most substituents with a lone pair adjacent to the ring, such as hydroxy, alkoxy, or acetamido. A small group of substituents, mostly the halogens, also direct substitution to the *ortho* and *para* positions, but retard the reaction, and so are designated as *ortho*, *para*-directing deactivators (OPDs). Finally, electron-withdrawing groups (EWGs) are designated *meta*-directing deactivators (MDs), since they retard the reaction and direct substitution primarily to the *meta* position.^4^

How a substituent affects the cationic intermediate elegantly rationalizes the behavior of the three types of substituents. This analysis is presented in all organic chemistry textbooks of which this author is aware.^1−3^ The OPAs are able to stabilize the intermediate through their electron-donating character but particularly strongly so when placed *ortho* or *para*. Only then does the positive charge end up on the same carbon atom as the substituent in at least one resonance form. The OPDs operate in a similar fashion, except that in these cases the substituents are sufficiently electron-withdrawing through the σ-system to destabilize the cationic intermediate while still stabilizing the *ortho* and *para* cases relative to *meta* via π-electron donation. Table 1 lists typical product distributions for reactions of benzene substituted with an OPA or an OPD, taken from the chemical literature.

**Table 1:** Representative Product Distributions for EAS Reactions on EDG-Substituted Benzenes^a^

	product distribution			relative partial rate factors		
	*ortho*	*meta*	*para*	*ortho*	*meta*	*para*
nitration						
bromobenzene	36.5%	1.2%	62.4%	0.18	0.01	0.62
chlorobenzene	29.6%	0.9%	69.5%	0.15	0.00	0.70
fluorobenzene	8.7%	0.0%	91.3%	0.04	0.00	0.91
toluene	58.4%	4.4%	37.2%	0.29	0.02	0.37
anisole	44.0%	0.0%	55.0%	0.22	0.00	0.56
chlorination						
bromobenzene	39.7%	3.4%	56.9%	0.20	0.02	0.57
chlorobenzene	36.4%	1.3%	62.3%	0.18	0.01	0.62
fluorobenzene	10.9%	0.0%	89.1%	0.05	0.00	0.89
toluene	74.7%	2.2%	23.1%	0.37	0.01	0.23
toluene (in acetonitrile)	37.6%	0.0%	62.4%	0.19	0.00	0.62
anisole	34.9%	0.0%	65.1%	0.17	0.00	0.65
anisole (in HOAc)	21.0%	0.0%	79.0%	0.11	0.00	0.79
acetanilide	32.5%	0.0%	67.5%	0.16	0.00	0.68
bromination						
anisole	1.6%	0.0%	98.4%	0.01	0.00	0.98
proton exchange (computational, based on stability of the cyclohexadienyl cation)^7^						
bromobenzene	26%	0%	74%	0.13	0.00	0.74
chlorobenzene	22%	0%	78%	0.11	0.00	0.78
fluorobenzene	6%	0%	94%	0.03	0.00	0.94
toluene	9%	0%	91%	0.05	0.00	0.91
anisole	2%	0%	98%	0.01	0.00	0.98

a Experimental data from ref (9)).

Finally, the MDs destabilize the cationic intermediate, but less strongly so when placed *meta* because in that case the positive charge avoids the carbon bearing the substituent in all major resonance forms. Molecular orbital arguments can also be used to explain the behavior of the various substituents, but these arguments are less frequently invoked, since they are more complex and ultimately lead to much the same conclusion.^5,6^Table 2 lists reactions of benzene substituted with an MD.

**Table 2:** Representative Product Distributions for EAS Reactions on EWG-Substituted Benzenes

		product distribution			relative partial rate factors		
	ref.	*ortho*	*meta*	*para*	*ortho*	*meta*	*para*
nitration							
benzonitrile	(9)	17.1%	80.7%	2.0%	0.09	0.40	0.02
	(8)	15–17%	81–83%	2%	0.08	0.41	0.02
benzoic acid	(4)	18%	80%	1%	0.09	0.40	0.01
	(8)	15–20%	75–85%	1%	0.09	0.41	0.01
ethyl benzoate	(4)	28%	68%	3%	0.14	0.34	0.03
	(8)	24–28%	66–73%	1–6%	0.13	0.35	0.04
benzaldehyde	(4)	18%	73%	9%	0.09	0.37	0.09
acetophenone	(8)	26%	72%	0–2%	0.13	0.36	0.01
nitrobenzene	(9)	6.4%	93.2%	0.3%	0.03	0.47	0.00
	(8)	5–8%	91–93%	0–2%	0.03	0.46	0.01
	(12)	7–10%	89–91%	1%	0.04	0.45	0.01
chlorination							
benzonitrile	(9)	23.2%	73.9%	2.9%	0.12	0.37	0.03
	(13)	34%	55%	11%	0.17	0.28	0.11
benzaldehyde	(4)	30%	64%	6%	0.15	0.32	0.06
nitrobenzene	(9)	17.6%	80.9%	1.5%	0.09	0.40	0.02
	(13)	24%	69%	7%	0.12	0.35	0.07
CF_3_benzene	(9)	15.7%	80.2%	4.1%	0.08	0.40	0.04
	(13)	9%	86%	4%	0.05	0.43	0.04
proton exchange (computational, based on stability of the cyclohexadienyl cation)							
benzonitrile	(7)	43%	55%	2%	0.22	0.28	0.02
benzoic acid	(7)	13%	87%	1%	0.06	0.43	0.01
methyl benzoate	(7)	18%	81%	1%	0.09	0.41	0.01
benzaldehyde	(7)	27%	72%	1%	0.14	0.36	0.01
acetophenone	(7)	32%	66%	2%	0.16	0.33	0.02
nitrobenzene	(7)	15%	85%	1%	0.07	0.42	0.01
nitrosobenene	(7)	30%	70%	0%	0.15	0.35	0.00
CF_3_benzene	(7)	6%	94%	0%	0.03	0.47	0.00
benzene SO_3_H	(7)	10%	89%	1%	0.05	0.45	0.00

Electronic structure calculations confirm that substituents stabilize or destabilize the cationic intermediates in the manner expected. Tables 1 and 2 list not only experimental results but also product distributions predicted from the relative stabilities of the cationic intermediates, as calculated using G4 electronic structure calculations with a simulated aqueous solvent.^7^ In this analysis, the intermediate is treated as the rate-determining transition structure at a reaction temperature of 25 °C. Basing the analysis only on the postulated intermediate is of course a simplification, and actual product distributions depend on other factors as well. For instance, the strength of the particular electrophile affects how early or late the transition structure is and thus how closely the transition structure resembles the intermediate. However, for many cases, the intermediate is a good enough model to serve as not just a qualitative but even as a semiquantitative guide.

What is not frequently discussed, but nonetheless well-known, is that the *ortho*, *para*-directors, both activators and deactivators, generally yield significantly more *para* product than *ortho* product.^8^Table 1 provides some representative examples from the literature. The preference for *para* is all the more substantial when one takes into account the fact that *ortho* substitution enjoys a 2:1 statistical advantage over *para*. The statistical preference for *ortho* can be factored out by considering partial rate factors, which reflect the rate constants at individual positions, i.e., an individual *ortho*, *meta*, or *para* carbon atom. From this perspective, the *para* position is at least three times as reactive as the *ortho* position for every case in Table 1 except for the nitration of toluene and anisole and one (seemingly anomalous) instance of the chlorination of toluene. In some cases, such as nitration and chlorination of fluorobenzene, bromination of anisole, and the computational results for fluorobenzene and anisole, reactivity favors *para* over *ortho* by a factor of 20 or more. Several factors contribute to the selectivity between *ortho* and *para*, first clearly enumerated by Norman and Taylor.^4^ Steric factors can play a role and are surely crucial with bulky groups such as *tert*-butyl. The substituent can also interact with the electrophilic reagent directly, and in fact, such interaction is sometimes used in synthesis to guide the reaction to the *ortho* position.

However, electronic factors also favor *para* substitution compared to *ortho*. Experimentally, this is evident from the fact that compact substituents such as fluorine, chlorine, and methoxy, all of which have small A-values (a common measure of steric size), nonetheless yield a substantial preference for *para* substitution. The preference can be rationalized in several ways: according to the charge distribution of the cyclohexadienyl cation (positive charge *para* > *ortho* > *meta*),^1,10^ or based on the fact that *para*-quinoid resonance forms are preferable to *ortho*-quinoid resonance forms,^1,7^ or using a perturbative MO argument.^7^ Electronic structure calculations of substituted cyclohexadienyl cations confirm the effect: EDGs placed *para* stabilize the cation more strongly than those placed *ortho*.^7,11^ Furthermore, the preference for *para* substitution is correlated with the strength of electron-donating character: the more strongly π-donating a substituent is, as measured by the Hammett σ^+^ parameter, the stronger the preference for *para* substitution.^7^

What appears never to have been highlighted, however, is the fact that MDs typically yield substantial amounts of *ortho* product as well as *meta* product and very little *para* product. Product distribution data for such cases is much more sparse than for the OPAs and OPDs, but Table 2 lists the data that could be readily found. The ratios of *ortho* to *meta* are not unlike the ratios of *ortho* to *para*, particularly if the latter are corrected for the statistical contribution, i.e., if one looks at the partial rate factors. The bottom entries in Table 2 indicate that the electronic structure calculations reveal the same effect. The *ortho* product typically constitutes at least 15% of the product mixture, and in many cases as much as 25–35% (e.g., nitration of ethyl benzoate and acetophenone, chlorination of benzonitrile and benzaldehyde, and the computational predictions for benzonitrile (43% *ortho*!), acetophenone, nitrosobenzene, and benzaldehyde).

This observation begs the question, why are EDGs designated as *ortho*, *para*-directors, while EWGs are designated simply as *meta*-directors, rather than as *ortho*, *meta*-directors? The answer, almost undoubtedly, is that the traditional arguments predict that the categories *should* be *ortho*, *para*-directing and *meta*-directing. We are always influenced by what we expect to see, and we see *ortho*, *para*-directors in the data from Table 1 and *meta*-directors in the data from Table 2 despite the fact that, in a purely objective sense, the relative favorability of *ortho* substitution is quite similar. Figure 1 emphasizes the point by graphically representing the typical product distributions for electrophilic aromatic substitution reactions on benzene substituted with either an *ortho, para*-director or a *meta*-director.

**Figure 1:**
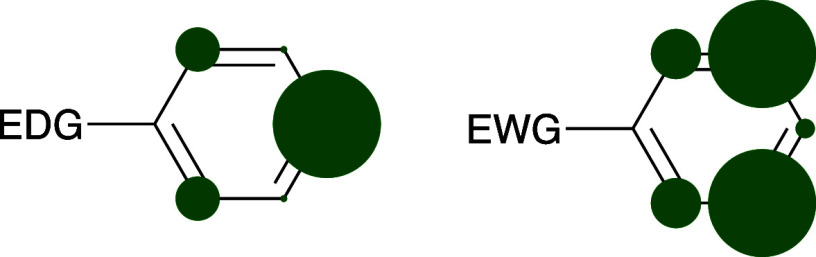
Graphical representation of the typical product distribution in the reaction of benzene substituted with either an EWG or an EDG. The areas of the green circles are proportional to the average amounts of products obtained at the sites marked. The data are taken from Tables 1 and 2. From Table 1, the average distribution is 28% *ortho*, 1% *meta*, and 71% *para*. From Table 2, the average distribution is 20% *ortho*, 77% *meta*, and 3% *para*.

There is, however, a rationalization for the substantial amount of *ortho* substitution in MD-substituted benzenes. Most simply, since the preference for *para* over *ortho* is correlated with electron-donating ability, there is some logic in the notion that for EWGs, *ortho* should be favored over *para*. But more satisfyingly, a perturbative MO analysis suggests that EWGs that are π-acceptors should preferentially be placed *ortho***or*****meta*** on a cyclohexadienyl cation. Figure 2 illustrates the key interaction in which the highest occupied molecular orbital (HOMO) of the cyclohexadienyl cation has a favorable interaction with the lowest unoccupied molecular orbital (LUMO) of the substituent. Due to the symmetry of the cyclohexadienyl cation HOMO, which has a node at the *para* carbon, the stabilizing interaction is only possible if the EWG is placed *ortho* or *meta*. The MO argument is put forth in more detail by Rablen and Yett.^7^

**Figure 2:**
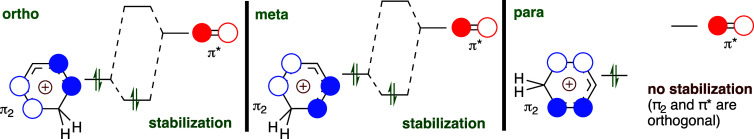
MO interactions between the cyclohexadienyl cation and a substituent that is an EWG and a π-acceptor. Favorable interactions are possible when the proton has been added *ortho* or *meta* to the pre-existing substituent but not when the proton is added *para* to the substituent, since π_2_ has a node at the *para* position.

This analysis assumes that the EWG has an empty, low-energy π* acceptor orbital. That is true, however, at least for most MDs: carbonyls and the cyano group, for instance. Furthermore, Table 2 shows that the extent of *ortho* substitution seems to be the most substantial for these traditional π-acceptors. And even groups such as trifluoromethyl, and perhaps sulfonic acid, can be thought of as π-acceptors in a hyperconjugative sense.

The MO argument is not meant to completely replace the classic argument based on charge distributions and resonance forms; that argument accounts for the significant preference for *meta* over *ortho* substitution that is reliably observed. But nonetheless, the MO argument provides a meaningful rationalization for the observation, based on both experiment and electronic structure calculations, that EWGs yield substantial amounts of *ortho* as well as *meta* product, but little *para* product.

This author would suggest that in thinking about the electrophilic aromatic substitution reaction, one should consider most EWGs to be *ortho*, *meta*-directing deactivators (OMDs) with a modest preference for *meta*, similar to how EDGs are considered *ortho*, *para*-directors with a modest preference for *para*.

## Data Availability

The data underlying this note are available in the published article.
